# Research on instrument mix and regional suitability of digital publishing industrial policies: An empirical exploration based on qualitative comparative analysis of fuzzy set

**DOI:** 10.1371/journal.pone.0346245

**Published:** 2026-04-13

**Authors:** Sirui Li, Johnny Fat Iam Lam

**Affiliations:** Faculty of Humanities and Social Sciences, Macao Polytechnic University, Macao, China; University of Kelaniya, SRI LANKA

## Abstract

Taking the digital publishing industry as its research object and employs the fuzzy-set qualitative comparative analysis (fsQCA) method to systematically examine the causal relationships among the Instrument Mix, regional conditions, and industrial performance, drawing on policy texts and development data from 16 major Chinese provinces. The findings indicate that neither a single Policy instrument nor an isolated regional condition is sufficient to sustain high-quality development; rather, industry success depends on specific configurations of multiple interacting conditions. Content application and scenario promotion, together with industry chain shaping and cluster development, form the core elements of multiple high-performance pathways. In contrast, fiscal and financial instruments play a relatively peripheral role, confirming both synergistic and substitutive relationships among Policy instruments. Further path analysis reveals three distinct realization logic in the digital publishing industry: (1) a regional environment–driven model relying on economic and cultural endowments; (2) a policy–economy dual-drive model characterized by joint government–market action; and (3) a systemic synergy model where multiple factors interact comprehensively. In contrast, non-high-performing configurations demonstrate causal asymmetry, indicating that industrial underperformance is not a simple inversion of success but rather the result of compensatory interactions among regional conditions.

## 1. Introduction

China’s digital publishing industry, as a key driver of the digital economy and a foundational component in constructing a culturally advanced nation, has exhibited rapid growth, with total output surpassing RMB 1.6 trillion in 2023 [1]. Notwithstanding this expansion, significant regional disparities are evident, as industrial resources remain concentrated in a limited number of developed provinces, resulting in a pronounced “centre–periphery” structure and presenting considerable policy challenges. In response, governments at various levels have promulgated a series of comprehensive policy documents—such as the Opinions on Promoting the Innovative Development of Cultural and Creative Industries—advocating for the coordinated deployment of a diverse array of policy instruments, including finance, taxation, industrial policy, innovation, and human resources. This strategy has led to the formation of a de facto policy instrument mix. Nevertheless, implementation outcomes of these policy combinations diverge markedly across regions, prompting the central research question: Do regional conditions determine the effectiveness of the policy instrument mix?

Although scholars have begun to address this issue, two critical research gaps remain. First, most studies take a “single-instrument” perspective. Some argue that financial subsidies stimulate enterprise innovation [2], tax incentives reduce market burdens [3], and copyright protection ensures market health. Yet, such analyses overlook that policies function in practice as integrated combinations of multiple instruments. Second, other studies [4,5] implicitly assume the universality of policy effectiveness. They neglect the principle of Regional Suitability. The same Instrument Mix may produce divergent outcomes across different regional contexts [6]. The constraints of traditional quantitative approaches, which cannot capture complex, multi-factor causal configurations, largely cause these limitations.

To address these gaps, this study adopts a configuration approach, moving beyond variable-centred reasoning. Policy Instrument Mix. Drawing on Howlett and colleagues [7],this study defines it as a holistic framework formed by the government’s organic integration of different Policy instruments to achieve industrial development objectives. Regional Suitability. Grounded in the Regional Innovation System (RIS) Theory, it is defined as the extent to which the effectiveness of a Policy Instrument Mix depends on its alignment with the embedded regional environment—that is, regional conditions such as economic base, industrial structure, and governmental capacity decisively shape policy performance. Accordingly, this study seeks to systematically examine how the Instrument Mix and regional conditions jointly drive the development of China’s digital publishing industry. Specifically, it addresses three core research questions: (1) what major types of Policy instruments exist in the current policy system of the digital publishing industry? (2) how do different Instrument Mixes influence industry development across regions? and (3) how do the Instrument Mix and regional conditions interact to generate differentiated development pathways? To this end, the study integrates policy text analysis with the fuzzy-set qualitative comparative analysis (fsQCA) method, systematically examining 16 provincial-level policy documents and regional development data to identify combinational patterns of Policy instruments and their Regional Suitability. The paper is structured as follows: Section 1 reviews the literature and presents the theoretical framework; Section 2 details the research methodology; Section 3 reports the empirical findings; Section 4 discusses the results; and Section 5 concludes with policy implications.

## 2. Literature review and theoretical foundation

### 2.1. The state of research on digital publishing industry policy

Research on digital publishing industry policy has grown substantially in recent years; however, it remains conceptually fragmented, divided between studies centred on Policy instruments and those emphasising regional dynamics. On the Policy instruments side, Chinese scholars have primarily focused on classification frameworks and optimization models. For example, Zhang Yao and Chu Peng (2021) classified China’s digital publishing policies into supply-side, demand-side, and environmental instruments based on Rothwell and Zegveld’s (1985) typology, highlighting the dominance of supply-side tools [8]. Wang Yue (2024) [9], on the other hand, observed that policy implementation remains fragmented, lacking systemic coordination. While several studies examine the impacts of individual Policy instruments, including copyright protection, fiscal subsidies, and tax incentives [10–13], most overlook the synergistic and substitutive interactions among multiple instruments. In the international literature, the Instrument Mix has emerged as a core analytical paradigm. Scholars such as Howlett, Cejudo, and Laranja contend that the effectiveness of an Instrument Mix depends on interactions among Policy instruments, which may yield either synergistic or conflicting outcomes [14–16]. For instance, in innovation policy, R&D subsidies implemented without adequate intellectual property protection may cause unintended knowledge spillovers, thereby diluting the intended policy impact. This underscores that the key question is not “whether instruments exist”, but rather “how they are combined”.

On the regional dimension, most studies draw upon the Regional Innovation System (RIS) framework. Asheim and Gertler (2005) [17] argue that innovation activities are deeply embedded in regional institutions and economic environments, and that policy effectiveness is highly contingent upon contextual conditions. Chinese scholars such as Hu et al. (2024) and Wang & Cen (2024) [18,19] emphasise that regional disparities in digital economy development and innovation capacity critically shape policy outcomes, which depend largely on economic foundations and governance capacity. However, most existing studies treat policy measures as homogeneous external factors, failing to examine the dynamic interplay between Policy instruments and regional contexts. International experiences further confirm the importance of alignment between Policy instruments and regional contexts. The EU’s “smart specialisation” strategy emphasises differentiated development paths based on regional endowments [20], while Nordic countries achieve a balance between innovation and social equity through well-coordinated Instrument Mixes [21]. Japan likewise stresses the dual importance of copyright protection and market guidance in its digital publishing policy framework [22].

In summary, the existing literature presents two main limitations: (1) domestic studies predominantly address individual policy instruments without systematically investigating the policy instrument mix; and (2) analyses of regional suitability remain at the macro level, largely disregarding the micro-level interactions between policy design and regional characteristics.

### 2.2. Theoretical framework and hypotheses

**Fig 1:**
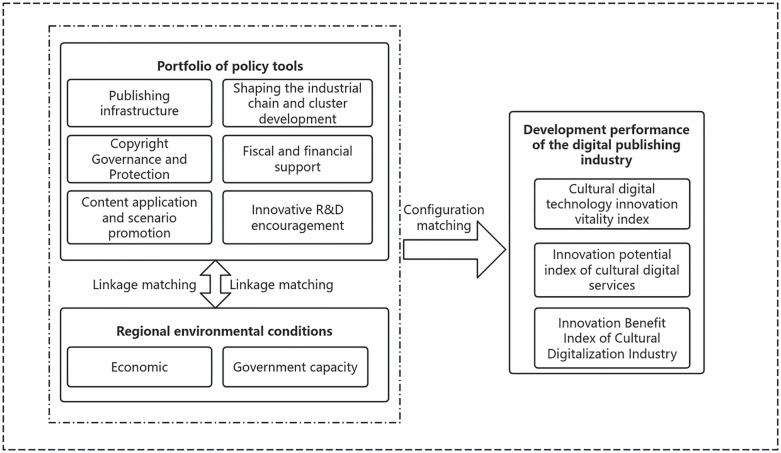
Digital publishing industry policy instruments policy-environment matching model.

This study integrates the Policy Instrument Mix Theory and the Regional Innovation System (RIS) Theory, constructing an analytical framework of “Policy Instrument Mix–Regional Environmental Conditions–Industrial Development Performance” (Fig 1) to uncover the complex causal mechanisms underlying digital publishing policy.

First, Policy Mix Theory forms the conceptual foundation of this study. It posits that the effectiveness of policy interventions addressing complex industrial challenges does not stem from isolated use of individual instruments but rather from the synergistic configuration of multiple complementary instruments [23,24]. The digital publishing industry spans multiple domains—technology, content, and markets, and thus requires multi-dimensional policy support. For example, the incentive effect of pure financial subsidies (a supply-based instrument) is greatly weakened in the absence of effective copyright protection (an environment-based instrument). Therefore, we propose the first hypothesis:

H1: The high performance of the digital publishing industry is not driven by any single Policy instrument but by a specific Policy Instrument Mix composed of multidimensional instruments.

To test this hypothesis, the study conducts content analysis of provincial policy texts, operationalising the Policy Instrument Mix into six core variables: (1) publishing infrastructure construction, (2) copyright governance and protection, (3) content application and scenario promotion, (4) industry chain shaping and cluster development, (5) fiscal and financial support, and (6) innovation and R&D encouragement.

Second, the Regional Innovation System (RIS) Theory provides a critical perspective for understanding variations in policy effectiveness. It emphasises that policy outcomes are highly context-dependent, deeply embedded in the intrinsic endowments of each region [25]. The regional economic base determines the market’s absorptive capacity and the efficiency of resource allocation, while government capacity influences the precision and effectiveness of policy implementation [26]. Together, these two conditions form the “institutional soil” necessary for effective policy execution. Therefore, we put forward the second hypothesis:

H2: The effectiveness of the Policy Instrument Mix is significantly moderated by regional economic fundamentals and government capacity, with multiple equivalent pathways capable of achieving high performance in alignment with Regional Suitability.

To evaluate Regional Suitability, this study identifies two core Regional Environmental Condition variables: economic fundamentals and government capacity. Finally, to comprehensively measure policy effectiveness, the study employs the Cultural Digital Innovation Index (CDI) as the outcome variable of digital publishing performance. The CDI integrates technological innovation, service innovation, and industrial efficiency, thereby systematically reflecting the high-quality development level of the industry.

## 3. Research methodology

### 3.1. Data sources

To ensure sample representativeness and data completeness, this study adopts a systematic three-stage sampling process. In the first stage, the initial scope was defined according to industrial foundation criteria. Based on the distribution of the first 70 “Digital Publishing Transformation Demonstration Units” announced by the former General Administration of Press and Publication (GAPP) in 2012, 20 provinces were identified as core areas for early-stage digital publishing development [27]. In the second stage, following the principle of regional balance, representative provinces from the above list were selected according to the National Bureau of Statistics’ regional classification (eastern, central, western, and northeastern China), ensuring coverage across different development levels and resource endowments. In the third stage, further screening was conducted based on data availability and accessibility. Digital publishing and related policy documents (2018–2022) were collected from provincial government portals and the Beida Faber database, while ensuring the availability of supporting data, such as the Cultural Digital Innovation Index (CDI), economic fundamentals, and government capacity indicators, from authoritative sources, including the China Statistical Yearbook. After this multi-stage screening, 16 provinces were ultimately selected as the final research sample (see Table 1). The objective of this study is to identify multiple policy combination pathways, rather than to generalise nationwide conclusions. Although the limited sample size constrains broad generalisation, it aligns well with the small- to medium-sized sample design of the fsQCA methodology, thereby enhancing its suitability for exploratory analysis of complex causal mechanisms.

**Table 1:** List of Digital Publishing Policies by Province.

Region	Province and city	Time	Release unit	Policy name
**Northeast China**	Jilin Province	2021	Jilin Provincial Government Affairs Service and Digital Construction Administration Bureau	“Digital Jilin” Construction Leading Group Office on the issuance of “Several Measures to Accelerate the Development of Jilin Province” notice
**East**	Shanghai Municipality	2018	The Shanghai Municipal Bureau of Press and Publication	Notice of Shanghai Municipal Press and Publication Bureau on the Issuance of the Three-year Action Plan for Starting the Brand of “Shanghai Publishing” (2018–2020)
	Beijing Municipality	2018	CPC Beijing Municipal Committee	Notice opinions on Promoting the innovative development of cultural and creative industries.
	Hebei Province	2020	The Peoples Government of Hebei Province	Digital Economy Development Plan of Hebei Province (2020–2025)
	Zhejiang Province	2018	Zhejiang Provincial Development and Reform Commission	Provincial Development and Reform Commission Provincial Party Committee Publicity Department on the issuance of “Zhejiang Province Cultural Reform and Development” 14th Five-year Plan “ notice
	a folk art form popular in Shandong	2021	Shandong Provincial Peoples Government	Notice of Shandong Provincial Peoples Government on printing and distributing the 14th Five-year Plan of Shandong Province
	Guangdong Province	2021	The People’s Government of Guangdong Province	Opinions of Guangdong Provincial Peoples Government on Accelerating Digital Development
	Hainan Province	2022	The General Office of the Hainan Provincial Peoples Government	The General Office of the Hainan Provincial Peoples Government On the printing of the Hainan provincial government digitization Notice of the Overall Transformation Plan (2022- −2025)
**Central section**	Henan Province	2021	The Peoples Government of Henan Province	Notice of the Peoples Government of Henan Province on printing and distributing the 14th Five-Year Plan of Henan Province
	Hubei province	2021	Hubei Provincial Peoples Government	Provincial people Government on printing and distributing Hubei Province digital economy development “fourteenth five-year plan” notice
	Jiangxi Province	2022	The People’s Government of Jiangxi Province	Notice of Jiangxi Provincial Peoples Government on printing and distributing the 14th Five-Year Digital Economy Development Plan of Jiangxi Province
**West**	Sichuan Province	2020	The General Office of the Provincial Party Committee, the General Office of the Provincial Government	The General Office of the CPC Provincial Committee and the General Office of the Provincial Government issued Guiding Opinions on Strengthening and Improving the Publishing Work and Promoting the High-quality Development of Publishing in Sichuan.
	Yunnan Province	2018	The General Office of the Yunnan Provincial Peoples Government	Yunnan Provincial Peoples Government on the issuance of Yunnan Province Further, expand and upgrade information consumption to release the potential of domestic demand implementation of the notice
	Xizang Autonomous Region	2018	The National Development and Reform Commission of the Tibet Autonomous Region	The Development plan of press, Publication, Radio, Film, and Television of Tibet Autonomous Region during the 13th Five-Year Plan period
	Shaanxi Province	2020	Shaanxi Provincial Department of Science and Technology	Shaanxi Provincial Department of Science and Technology and six other departments issued the Notice of the Implementation Opinions of Shaanxi Province on Promoting the Deep Integration of Culture and Science and Technology
	Qinghai Province	2021	The General Office of the Qinghai Provincial Peoples Government	The General Office of the Peoples Government of Qinghai Province issued the 14th Five-Year Plan and Plan for National Economic and Social Development of Qinghai Province

### 3.2. Fuzzy-set qualitative comparative analysis (fsQCA)

This study aims to examine how multiple concurrent policy and environmental factors jointly drive the development of the digital publishing industry, representing a typical configurational problem. Traditional regression-based approaches focus on isolating the independent net effects of variables, making it difficult to capture the complex interactions and substitutions among multiple factors in reality. Fuzzy-set Qualitative Comparative Analysis (fsQCA), grounded in set theory and configurational logic, identifies combinations of sufficient and/or necessary conditions leading to specific outcomes, rather than the isolated effects of single variables [28]. Its key advantage lies in recognising “equifinality”—that is, multiple distinct configurational paths can lead to the same outcome [29]. The fsQCA approach provides a theoretical and empirical foundation for understanding why differentiated Instrument Mix configurations are necessary across regions to achieve shared development objectives.

The fsQCA approach is particularly suitable for medium-sized sample studies, balancing case-specific depth and cross-case comparability. This study employs provincial administrative units as the analytical level, with a sample size of 16, squarely within the small- to medium-sized range. Under such sampling conditions, the distributional assumptions and degrees of freedom required by traditional large-sample statistical methods are difficult to meet, often overlooking case-specific heterogeneity. In contrast, fsQCA integrates the advantages of systematic comparison and holistic case understanding, constituting a “case-oriented” quantitative methodology [30]. This methodology uncovers the multiple causal pathways involving Policy instruments and Regional Suitability, ensuring both contextual specificity and cross-case generalisability, making it highly compatible with the research objectives of this study.

### 3.3. Variable design and measurement

#### 3.3.1. Definition of the outcome variable.

The digital publishing industry exhibits both knowledge and technology-intensive characteristics, with development performance reflected not only in economic output, but also in technological innovation, service transformation, and industrial ecosystem optimisation [31]. As a single economic indicator cannot fully capture the multi-dimensional nature of high-quality development, this study adopts the Cultural Digital Innovation Index (CDI) as the core outcome variable. The CDI is derived from the Research Report on China’s Cultural Digital Innovation Index (2023), which encompasses three dimensions: technological innovation vitality, service innovation potential, and industrial innovation benefits systematically corresponding to the digital publishing industry’s core performance in technology application, industrial evolution, and market value. The index addresses the limitations of traditional financial indicators by adopting a standardised measurement system, thereby enabling cross-regional comparison based on provincial panel data(see Table 2) [32]. Although developed within the Chinese context, the CDI’s design is conceptually aligned with the OECD Cultural Industry Indicator System (2022) [33], both emphasising composite measurement across technological, service, and industrial dimensions. It also corresponds closely to the EU Creative Industries Innovation Index [34]. Therefore, the CDI is suitable for inter-provincial comparison and as an internationally comparable indicator of cultural innovation performance.

**Table 2:** Cultural Industry Development Index of Chinese provinces and cities in 2023.

Provinces and Cities	Cultural Digital Innovation Index score
Beijing Municipality	87.82
Hebei Province	70.84
Jilin Province	73.22
Shanghai Municipality	80.54
Zhejiang Province	79.73
a folk art form popular in Shandong	75.19
Henan Province	72.41
Hubei province	75.41
Jiangxi Province	73.04
Guangdong Province	86.16
Hainan Province	75.98
Sichuan Province	75.53
Yunnan Province	71.15
Xizang Autonomous Region	73.43
Shaanxi Province	74.15
Qinghai Province	69.61

#### 3.3.2. Design of conditional variables.

The conditional variables are designed to systematically examine the interactive effects of policy interventions and regional endowments, categorised into two dimensions: Policy instruments and regional environment. The categorisation of Policy instruments draws on Rothwell and Zegveld’s (1985) tripartite framework of supply, environment, and demand-oriented instruments [35], but this study refines it into six operational variables reflecting China’s digital publishing practice: publishing infrastructure construction (SJBSS), copyright governance and protection (BQZL), content application and scenario promotion (NRYY), industry chain shaping and cluster development (CYSC), financial and fiscal support (CZJR), and innovation and R&D encouragement (YFGL). Each variable was measured based on provincial policy documents (2018–2022). Using paragraphs as coding units, the study conducted content analysis and applied relative frequency, rather than absolute counts, to control for textual length variations. Semantic repetitions were counted only once to ensure independence and coding validity (see Appendix 1 for coding procedures).

The regional environment variables reflect the contextual conditions under which policies operate, comprising economic base and government capacity dimensions. The economic base is measured using GDP per capita and per capita disposable income (PCDI) data from the China Statistical Yearbook. Government capacity is assessed via the Balanced Assessment Report on Digital Government Capacity (2023), incorporating digital resource access (ADR), digital support capacity (DSC), and digital empowerment capacity (DEC) indicators [36]. For fsQCA calibration, all continuous variables were transformed into fuzzy-set membership scores using the direct calibration method, applying the 75%, 50%, and 25% quartiles as anchors for full membership, crossover, and non-membership, respectively. Inter-coder reliability was assessed via Cohen’s Kappa, with all values exceeding 0.80 (SJBSS: 0.85, BQZL: 0.82, NRYY: 0.88, CYSC: 0.81, CZJR: 0.83, YFGL: 0.86), indicating “almost perfect” agreement [37].

### 3.4. Data calibration

The fsQCA procedure requires calibrating raw data into fuzzy-set membership scores ranging from 0 (full non-membership) to 1 (full membership). In this study, the direct calibration method was implemented in fsQCA 4.0, with the 75th, 50th, and 25th percentiles of each variable serving as qualitative anchors for full membership (0.95), crossover (0.5), and full non-membership (0.05) [28], a common approach in small- and medium-sized fsQCA applications [30]. The specific calibration thresholds for each variable are reported in Table 3. Following calibration, a necessity analysis was conducted to identify individual conditions required for the outcome, adopting a consistency threshold of 0.90, as commonly recommended in the literature. Subsequently, a truth table was constructed and normalised. Following Du Yunzhou and Jia Liangding (2017) [38], the raw consistency threshold was set at 0.80 and the case frequency threshold at 1, balancing analytical rigour and generalisability. Boolean minimisation procedures were then performed, primarily reporting intermediate solutions that incorporated reasonable logical remainders. Core and peripheral conditions were distinguished by comparing intermediate and parsimonious solutions. Finally, the adequacy and explanatory power of each configurational path were evaluated through consistency and coverage indicators, ensuring the transparency and robustness of the analytical results.

**Table 3:** Variable definitions and calibration anchors.

Variable type	Variable name	Variable description	Data source/measurement method	Calibration anchor point		
				Fully affiliated (0.95)	Intersection (0.5)	Fully unaffiliated (0.05)
**Outcome variables**	CDI	Cultural Digital Innovation Index	Research Report on China’s Cultural Digital Innovation Index (CDI) (2023)	80.54	75.19	71.15
**Conditional variables (Policy instruments)**	SJBSS	Frequency of publication infrastructure development	Content analysis of policy texts (paragraph counts)	5.25	3.00	1.75
	BQZL	Frequency of copyright governance and protection		5.25	2.50	1.75
	NRYY	Frequency of content application and scenario promotion		6.75	4.00	1.75
	CYSC	Frequency of industry chain shaping and cluster development		7.75	4.50	3.00
	CZJR	Frequency of financial and monetary support		3.25	1.50	1.00
	YFGL	Frequency of innovation and R&D incentives		6.75	4.50	3.00
**Conditional variables (regional environment)**	PCDI	Disposable income per capita (yuan)	China Statistical Yearbook (2022)	49327	33192	29797
	GDP	Total GDP (billion yuan)		60132.9	43944.1	13531.19
	ln(GDP)	(Natural logarithm of GDP, used to calculate quartiles)	(calculated from GDP)	*11.00*	*10.69*	*9.51*
	ADR	Digital Resource Access Capacity	Digital Government Capacity Equalisation Assessment Report (2023)	28.78	26.85	22.04
	DSC	Digital Support Capacity		32.22	29.72	23.63
	DEC	Digital Enabling Capabilities		33.34	27.77	24.44

Note: Calibration anchors are calculated based on the actual data distribution of the 16 sample provinces. The median of the Policy instruments frequency variable was calculated by interpolation to accurately reflect the relative position of the cases.

## 4. Research findings

### 4.1. Descriptive analysis

**Fig 2:**
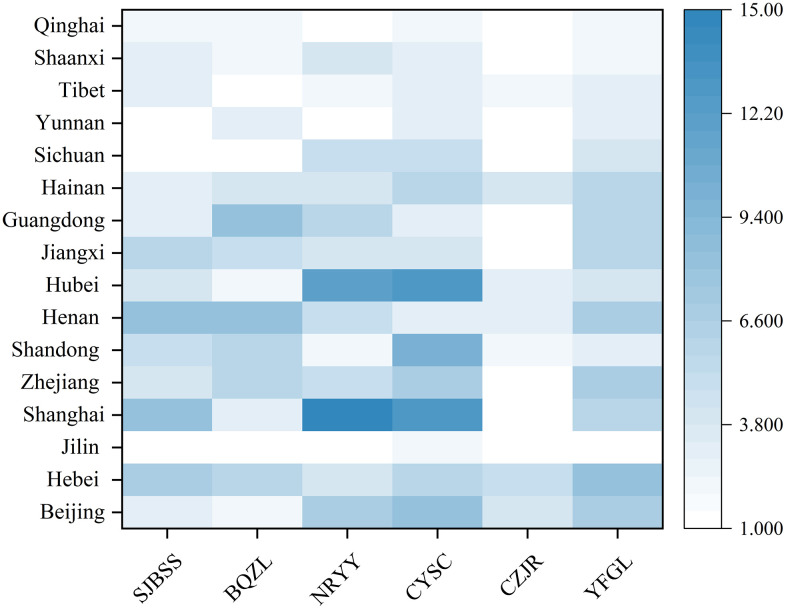
Heat map used by provincial policy tools.

Through a systematic analysis of Policy instrument usage frequency, this study finds that their application exhibits distinct structural characteristics (Fig 2). In terms of usage intensity, industry chain shaping and cluster development (CYSC) emerges as the most dominant Policy instrument, accounting for 22.9%. This is followed by content application and scenario promotion (NRYY) and innovation and R&D encouragement (YFGL), which account for 19.6% and 18.8%, respectively. In contrast, the Fiscal and Financial Support category exhibits the lowest frequency of Policy instrument utilization, accounting for only 8.0%. Taken together, the cumulative application of the three Policy instruments of industrial clusters, content application, and innovation and R&D reaches 61.3%, underscoring the strategic focus of governmental policy orientation.

Regional disparities are also pronounced, with eastern provinces generally demonstrating a more balanced and comprehensive application of various instruments, whereas western and northeastern regions remain relatively weak in infrastructure and fiscal support. The western and northeastern regions exhibit the weakest performance in both infrastructure development and fiscal–financial support. Specifically, Sichuan, Yunnan, and the Tibet Autonomous Region show almost no application of these two types of Policy instruments. Conversely, in the eastern region, the implementation of publishing infrastructure development is more advanced—particularly in Shanghai, Henan, and Hebei, which exhibit stronger development momentum. By contrast, development in western provinces such as Sichuan, Yunnan, and Qinghai remains insufficient. To enable further analysis, this study coded and summarized policy documents following the methodological procedures of fuzzy-set qualitative comparative analysis (fsQCA) and converted the variables into membership scores to construct a truth table for subsequent analysis.

### 4.2. Necessity analysis of single variables

Necessity analysis serves as a preliminary step to assess whether any single conditional variable is necessary for the outcome to occur [29]. The results clearly indicate that no single necessary condition exists. As shown in Table 4, the consistency levels of all individual conditions fall below the accepted 0.9 threshold, implying that no single factor, whether a Policy instrument or regional endowment, determines industrial success. This finding refutes the notion of single-factor determinism and directly supports the core hypothesis of this study: that the high-quality development of the digital publishing industry arises not from any single advantage but from specific configurations of multiple conditions. Specifically, although the consistency scores for Digital Enabling Capability (DEC) and Per Capita Disposable Income (PCDI) are relatively high (0.901 and 0.898, respectively), subsequent configurational analyses show that some high-performance paths exclude these conditions, indicating that they are not strictly necessary. Detailed results are presented in Table 4.

**Table 4:** Necessity analysis of individual condition variables.

Variable classification	Condition variable	High culture of digital innovation		Condition variable	Non-high-cultural digital innovation	
		Consistency	Fraction of coverage		Consistency	Fraction of Coverage
**policy instrument**	SJBSS	0.695055	0.570462	SJBSS	0.569954	0.560316
	~SJBSS	0.464286	0.474053	~SJBSS	0.563073	0.688640
	BQZL	0.554945	0.528796	BQZL	0.477064	0.544503
	~BQZL	0.521978	0.454545	~BQZL	0.587156	0.612440
	NRYY	0.844780	0.711806	NRYY	0.402523	0.406250
	~NRYY	0.295330	0.292120	~NRYY	0.714450	0.846467
	CYSC	0.784341	0.750329	CYSC	0.313073	0.358738
	~CYSC	0.329670	0.286055	~CYSC	0.782110	0.812872
	CZJR	0.776099	0.512704	CZJR	0.769495	0.608893
	~CZJR	0.407967	0.596385	~CZJR	0.384174	0.672691
	YFGL	0.682692	0.634738	YFGL	0.417431	0.464879
	~YFGL	0.424451	0.378213	~YFGL	0.672018	0.717258
**economic base**	PCDI	0.898352	0.842783	PCDI	0.334862	0.376289
	~PCDI	0.335165	0.296117	~PCDI	0.860092	0.910194
	GDP	0.747253	0.668305	GDP	0.378440	0.405405
	~GDP	0.335165	0.310433	~GDP	0.690367	0.765903
**Digital government capability**	ADR	0.751374	0.708549	ADR	0.370413	0.418394
	~ADR	0.383242	0.336957	~ADR	0.741973	0.781401
	DSC	0.872253	0.772506	DSC	0.346330	0.367397
	~DSC	0.285714	0.267352	~DSC	0.785550	0.880463
	DEC	0.901099	0.843188	DEC	0.344037	0.385604
	~DEC	0.343407	0.304136	~DEC	0.860092	0.912409

Note: ~ indicates the logical error, no ~ before the variable indicates that the variable is in the existence state, and ~ indicates that the variable is missing.

### 4.3. Analysis of configurational pathways

Based on the intermediate solution results (Table 5), this study identifies six configurational pathways (P1–P6) that constitute sufficient conditions for achieving high-level digital innovation in the cultural sector. The consistency levels of all pathways exceed 0.93, with an overall solution consistency of 0.98, indicating that the model demonstrates strong explanatory validity. Based on the compositional characteristics of core conditions, these six pathways can be grouped into three prototypical driving models: (1) Regional environment-driven model (Path P1). The core logic of this pathway lies in leveraging a region’s inherent endowments and contextual advantages. Rather than relying on large-scale infrastructure investment or content promotion, it transforms high levels of per capita disposable income (PCDI) and governmental digital support capacity (DSC) into industrial advantages through industry chain shaping (CYSC) and R&D encouragement (YFGL). Hainan Province serves as a representative case of this pathway. (2) Policy–economy dual-driven model (Paths P2 and P3). This configuration is characterised by the synergistic interaction between a strong economic foundation (high GDP) and key Policy instruments. Specifically, Path P2 emphasises market activation through content application promotion (NRYY), whereas Path P3 focuses on institutional optimisation via copyright governance (BQZL). Provinces such as Guangdong, Shandong, Shanghai, and Zhejiang exemplify the operational logic of this model. (3) Systemic synergy model (Paths P4–P6). This pathway demonstrates a multi-dimensional and comprehensive alignment among Policy instruments, economic fundamentals, and governmental capacity. In these configurations, core Policy instruments—including content application (NRYY) and industry chain shaping (CYSC)- align closely with regional conditions such as high income levels and robust digital governance capacity, thereby forming a systematic synergy that drives the industry toward advanced development.

**Table 5:** Organisational pathways for digital innovation in high culture.

Conditional variable		Digital innovation for high culture					
		Regional environment-driven	Digital economy tool-driven		Fully coordinated		
		P1	P2	P3	P4	P5	P6
**Policy instruments**	**SJBSS**	⊗	●	●	⊗	⊗	⊗
	**BQZL**	●	⊗	●	⊗	●	⊗
	**NRYY**	⊗	●	⊗	●	●	●
	**CYSC**	●	●	●	●	⊗	●
	**CZJR**	●	●	●	●	⊗	⊗
	**YFGL**	●	⊗	⊗	●	●	⊗
**Economic foundation**	**PCDI**	●	●	●	●	●	⊗
	**GDP**	⊗	●	●	⊗	●	●
**Government capacity**	**ADR**	⊗	●	⊗	●	●	⊗
	**DSC**	●	⊗	●	●	●	●
	**DEC**	⊗	⊗	●	●	●	●
**Consistency**		0.97	0.95	0.95	1	1	1
**Raw Coverage**		0.08	0.12	0.10	0.10	0.09	0.09
**Unique Coverage**		0.06	0.08	0.07	0.06	0.06	0.07
**Case Province**		Hainan	Guangdong, Shandong, Sichuan		Henan, Shandong, Shanghai, Zhejiang, Hubei		
**Solution Consistency**		0.98					
**Solution Coverage**		0.60					

Note: A large ‘●’ represents the presence of a core condition, a small ‘●’ represents the presence of a marginal condition, a large ‘⊗’ represents the absence of a core condition, a small ‘⊗’ represents the absence of marginal conditions, and a blank indicates that the condition variable is not significant for the outcome.

Additionally, the analysis yields two notable findings: (1) all high-performance configurations include at least one core condition, either economic fundamentals (PCDI or GDP) or governmental capacity (DSC or DEC), and none rely solely on Policy instruments without the backing of Regional Suitability; (2) the Fiscal and Financial Support instrument (CZJR) functions as a secondary condition in most high-performance configurations (P1–P4) and is absent in some (P5, P6), suggesting that it does not directly drive industrial innovation.

In contrast, pathways associated with low industrial performance reveal a pattern of causal asymmetry. As shown in Table 6, the three non-high-performance configurations (NP1–NP3) are generally characterised by a dual deficiency in economic fundamentals and governmental capacity, with typical cases observed in Yunnan and Qinghai, which remain relatively underdeveloped. For example, Yunnan and Qinghai both exemplify this “dual-deficit” configuration. In these regions, even when governments implement certain Policy instruments (e.g., the presence of CYSC in the NP2 pathway, Table 6), these measures remain ineffective at the industrial level due to inactive market participation (weak economic base) and insufficient digital governance capacity (weak government institutions).

**Table 6:** Organisational pathways for digital innovation in non-high culture.

Conditional variable		Digital innovation in non-high culture		
		Economic-Governmental Regional Environment Deficit Type		
		NP1	NP2	NP3
**Policy instruments**	**SJBSS**	●	●	●
	**BQZL**	●	●	●
	**NRYY**	⊗	⊗	●
	**CYSC**	⊗	●	⊗
	**CZJR**	⊗	●	●
	**YFGL**	⊗	●	●
**Economic foundation**	**PCDI**	⊗	⊗	⊗
	**GDP**	⊗	⊗	●
**Government capacity**	**ADR**	⊗	⊗	●
	**DSC**	⊗	●	⊗
	**DEC**	⊗	⊗	⊗
**Consistency**		1	0.83	0.96
**Raw Coverage**		0.07	0.12	0.08
**Unique Coverage**		0.04	0.06	0.06
**Case Province**		Jiangxi, Yunnan, Jilin, Tibet, Qinghai		
**Solution Consistency**		0.95		
**Solution Coverage**		0.56		

Note: A large ‘●’ represents the presence of a core condition, a small ‘●’ represents the presence of a marginal condition, a large ‘⊗’ represents the absence of a core condition, a small ‘⊗’ represents the absence of marginal conditions, and a blank indicates that the condition variable is not significant for the outcome.

### 4.4. Robustness checks

To verify the robustness and reliability of the research findings, this study conducts a sensitivity analysis by adjusting the calibration and consistency thresholds. First, the original calibration anchors of 75%, 50%, and 25% were adjusted to 70%, 50%, and 30%, respectively. After re-analysis, the core conditions across pathways remained stable, with only minor changes in peripheral conditions (e.g., “~BQZL” shifting from peripheral to core in Path P2). The overall logical structure and case-matching relationships remained unchanged. Second, the consistency threshold was increased from 0.80 to 0.85. The results indicate that pathways with lower coverage (e.g., P5) were excluded, while P1–P4 and P6 remained stable. The core conditions remained unchanged, and the overall solution consistency and coverage remained above 0.95 and 0.55, respectively. These results demonstrate that the three types of pathways, regional environment-driven, digital economy tool-driven, and comprehensive coordination models, exhibit strong robustness and cross-configuration stability.

## 5. Discussion

### 5.1. Instrument mix effects and substitution relationships of core policy instruments

The results of the configurational analysis reveal that the Policy Instrument Mix promoting the high-quality development of the digital publishing industry exhibits a distinct “core–periphery” structure. Content application and scenario promotion (NRYY) and industry chain shaping and cluster development (CYSC) constitute the stable core of the Instrument Mix within high-performance pathways. This finding supports the central proposition of Policy Mix Theory regarding the synergistic effects of an Instrument Mix [39], further revealing that the essence of synergy lies in the dynamic coupling between “demand pull” and “supply upgrading.” Content application instruments directly stimulate industrial innovation by expanding markets and generating revenue, whereas industry chain shaping instruments provide a structural foundation for innovation by optimising the industrial ecosystem and facilitating knowledge spillovers. The effective interaction between the two fosters a virtuous cycle in which supply creates demand and demand, in turn, stimulates supply.

The practices of Beijing and Sichuan Provinces jointly illustrate the fundamental role of this core Instrument Mix. Leveraging its status as a national cultural centre, Beijing has vigorously expanded content application scenarios (NRYY) through initiatives such as the “Book-Scented Beijing” all-media reading platform and community-based “Digital Reading Stations” [40]. Simultaneously, through the systematic development of industrial clusters such as the National Digital Publishing Base (CYSC), Beijing has effectively promoted the spatial concentration of technology, capital, and creative talent. Sichuan Province has demonstrated the adaptability of this policy combination in regions with distinct resource endowments. Through the “Revitalising Sichuan Publishing” strategy, the province has integrated scenario-based initiatives such as “digital reading in schools” (NRYY) with the intensive development of key publishing units, cultural and creative industrial parks, and other industry chain initiatives (CYSC) [41]. The successful experiences of both provinces demonstrate that the synergy between “scenario development” and “ecological construction” constitutes a common mechanism driving digital publishing innovation.

In contrast, financial instruments (CZJR) play a marginal or supplementary role in most high-performance configurations. This finding does not diminish the importance of financial inputs; rather, it highlights that their effectiveness in the digital publishing industry, a sector driven by creativity and human capital, is highly contingent upon its institutional and market environment. The case of Hubei Province provides further empirical insight into this relationship. Although the province has established special funds for the cultural industry, its key policy innovation lies in the creation of the “Hubei Provincial Platform Service System for Promoting Digital Publishing,” which significantly enhances the policy impact of limited fiscal resources through project matching, technical support, and market information services [42]. This demonstrates that the function of fiscal instruments is not a one-way supply mechanism, but should rather focus on “catalysing” and “leveraging” market and social capital. As crowdfunding, venture capital, and other market-oriented financing mechanisms become increasingly mature, the role of direct government subsidies is shifting from “primary input” to “credit enhancement” and “market gap compensation.” This transformation aligns with the “enabling industrial policy” framework proposed by Rodrik [43], which emphasises that governments should guide industrial development through institutional construction and ecosystem creation, rather than relying on direct subsidies or unilateral intervention.

### 5.2. Multiple paths of realisation of regional innovation systems

The results of the configurational analysis support the assertion of the Regional Innovation System theory that policy effectiveness is context-dependent [17], thereby revealing that high performance can be achieved through multiple, context-specific pathways.

The regional environment-driven pathway (P1) exemplifies the logic of endowment dependence. Hainan’s per capita disposable income ranks in the middle-to-upper range nationwide, while the government’s leadership and coordination capacity in digitalisation strategies, as reflected in the Outline of the 14th Five-Year Plan for the Development of the National Economy and Society of Hainan Province and the Vision for 2035, constitutes a unique advantage in its Digital Support Capacity (DSC). In terms of Policy instruments, the P1 pathway places core emphasis on industry chain shaping, copyright governance, and financial R&D incentives, while being less reliant on infrastructure investment (SJBSS) and content scenario promotion (NRYY). This configuration is highly consistent with Hainan’s policy practice. The province has established a Digital Publishing Industrial Park and issued the Hainan Province Financial Measures for Promoting High-Quality Economic Development to subsidise R&D and innovation. However, the construction of its digital publishing public platform (SJBSS) remains relatively underdeveloped, and its limited market capacity constrains the effectiveness of large-scale content scenario promotion [44]. Therefore, Hainan’s success does not derive from comprehensive coverage but from the precise transformation of regional endowments into industrial dynamism. By focusing on industrial ecology and innovation incentives, this pathway embodies the essence of P1, confirming Storper’s thesis of “regional specificity”—that development must be grounded in local endowments [45].

Policy–Economy Dual-Driven Pathways (P2/P3). Under the common framework of “government-led” development, two differentiated logics have evolved. The P2 pathway (represented by Guangdong) can be characterised as “market-driven.” At its core, it relies on a mega-regional economy (GDP of RMB 13.57 trillion in 2023) and prioritises the activation of market demand through scenario development. For instance, Guangdong’s digital publishing industry leverages its vast Internet user base of over 150 million to promote the integration of “digital reading + education” and “digital reading + governance.” The provincial national reading index averages 66.47 [46], reflecting how market dynamics directly stimulate industrial development.

In contrast, the P3 pathway (represented by Shandong) exemplifies a “governance-driven” approach. While leveraging its position as a major economic province, Shandong places greater emphasis on establishing industrial order through institutional supply. Shandong has established a “government–industry–academia–research–finance” innovation and entrepreneurship community and developed a Shandong Copyright Management and Service Platform, which recorded over 1.5 million copyright registrations between 2022 and 2023, reflecting a year-on-year increase of approximately 30% [47], providing an institutional platform for digital content creation and transactions. A comparison of the two provinces demonstrates that, although both are economic–policy-driven, distinct regions can achieve high-quality development through differentiated Instrument Mix configurations. This finding aligns with Asheim and Gertler’s (2005) argument on the context dependence of Regional Innovation Systems, indicating that equifinal pathways are heterogeneous and that regional endowments play a more decisive role.

The systemic synergy pathways (P4–P6) are characterised by the comprehensive integration of economic foundations, Policy instruments, and governmental capabilities. Shanghai has pursued parallel efforts in content promotion (NRYY) and industry chain clustering (CYSC). Its online literature sales revenue reached RMB 12 billion in 2022 [48]. Combined with its high per capita income and comprehensive digital governance capacities (ADR, DSC, DEC), these factors have formed a solid innovation synergy. Zhejiang, meanwhile, has achieved a dual circulation of online and offline publishing through its “1+4+N” Smart Publishing System, which facilitates full-chain interaction within the publishing industry. The experiences of these two provinces indicate that high performance arises from the systemic synergy of multiple dimensions, rather than reliance on any single advantage. This evolutionary logic is consistent with Teece’s (2018) theory of dynamic capabilities [49], which posits that sustainable competitive advantage derives from the continuous reconfiguration of multiple factors within institutional frameworks.

### 5.3. Causal asymmetry and the boundaries of industrial policy

One of the central findings of this study is “causal asymmetry”, that is, the pathway leading to failure is not merely the inverse of that leading to success. This finding offers a novel analytical perspective for understanding the evolving boundary between government and market in contemporary industrial policy theory.

The core characteristic of the non-high-performing pathways (NP1–NP3) is a “double deficit” in both economic fundamentals and governmental capacity. This finding suggests that industrial policy is not omnipotent; its effective implementation requires two fundamental prerequisites: a market with basic purchasing power and a government with adequate digital governance capacity. When these foundational conditions are absent, any upper-level Policy instruments resemble rootless trees, hardly effective. This observation underscores that the primary role of government is that of an “enabler” to create and sustain a healthy market and institutional environment. However, once this “survival threshold” is surpassed, the logics driving success become diversified, reflecting equifinality rather than linear causation. The success of regional environment-driven (P1), policy–economy-driven (P2/P3), and systemic synergy (P4–P6) pathways indicates that the government’s role evolves from an “enabler” to a “catalyst.” The government no longer needs to, nor should it pursue a single “optimal solution.” Instead, it should leverage its specific regional endowments to catalyse industrial development across diverse dimensions.

The case of Jilin Province serves as an illustrative example of this non-linear policy mechanism. Jilin exhibits low affiliation scores across most conditions, positioning it within the non-high-performing cluster (NP1). However, its CDI score of 73.22 exceeds that of several provinces with more favourable economic conditions, such as Yunnan (71.15). A deeper examination reveals that, despite overall weakness, Jilin’s Digital Support Capacity (DSC) ranks in the mid-to-high range, exerting a critical “compensation effect.”This suggests that a well-functioning digital government platform or efficient public data service system—the institutional embodiment of DSC—can provide critical institutional support and information resources for enterprises when the market lacks strength. Such mechanisms partially offset the disadvantages of a small market (~PCDI) and weak economic vitality (~GDP), thereby validating the core premise of configurational group theory [50]: the impact of any single condition depends on the specific configuration in which it is embedded, and a moderately strong advantage can exert a non-linear yet decisive positive effect within a combination of otherwise weak conditions.

## 6. Conclusions and implications

### 6.1. Research conclusion

This study analyses the configurational effects of digital publishing industrial policies using the fsQCA method, which not only verifies the initial hypotheses but also yields new insights at both theoretical and methodological levels. First, the core theoretical contribution lies in revealing the complex causal mechanisms underlying industrial policy effects. By confirming the absence of any single necessary condition and identifying three equifinal pathways, namely regional environment-driven, policy–economy dual-driven, and systemic synergy, this study transcends the traditional “single optimal solution” paradigm, providing empirical support for the principle of equifinality within Policy Mix Theory. Moreover, by examining the causal asymmetry between successful and unsuccessful pathways, this study addresses contemporary debates on the boundary between government and market, revealing the dynamic evolution of the government’s role from a basic “enabler” to an advanced “catalyst.”Additionally, the identification of a “compensation mechanism” (as exemplified by Jilin Province) extends the Regional Innovation Systems (RIS) framework, demonstrating that regions can enhance resilience through asymmetric advantages, rather than merely compensating for all deficiencies. Second, at the methodological level, this study demonstrates the analytical value of fsQCA in policy research concerning cultural and digital industries. The method effectively captures multifactorial and concurrent configurational effects, providing a powerful analytical instrument that complements traditional approaches to understanding policy phenomena in complex, small-to-medium-sized samples. Naturally, this study has limitations—particularly regarding the quantification of policy intensity. Future research could employ longitudinal case tracking or process-tracing methods to elucidate the dynamic evolution mechanisms of Policy instrument combinations and Regional Suitability across time.

### 6.2. Policy implications

Based on the above findings, this paper puts forward the following policy recommendations:

First, implement differentiated regional policy strategies. Policymaking should abandon the “one-size-fits-all” approach and instead pursue precision-based strategies grounded in regional endowments. For environmentally driven regions (e.g., Hainan), policies should focus on transforming inherent advantages into dynamic capabilities. Rather than pursuing comprehensive development indiscriminately, resources should be concentrated on maximising unique locational or cultural advantages through industry chain shaping (CYSC) and R&D incentives (YFGL), thereby building distinctive industrial ecosystems with pronounced competitive “long boards.”For dual-driven regions (e.g., Guangdong and Henan), the core Instrument Mix should be tailored to their dominant logic, whether market-driven or governance-driven. Specifically, market-driven regions should prioritise industrial chain upgrading through the construction of major application scenarios, while governance-driven regions should focus on institutional supply, optimising the business environment for copyright protection and data governance.

Second, optimise the application and coordination of Policy instruments. The findings indicate that content application, scenario promotion, and industry chain shaping and cluster development constitute the core of the Policy Instrument Mix. For policy–economy dual-driven regions (e.g., Guangdong and Shandong), the use of these two Policy instruments should be reinforced to drive industrial chain upgrading through major scenario construction, reducing reliance on direct fiscal inputs, and increasingly mobilising market mechanisms to attract social capital. Simultaneously, regional co-development mechanisms, such as enclave economies and industrial collaboration frameworks, should be established to alleviate resource constraints and enhance cross-regional integration.

Finally, establish a dynamic policy adaptation and adjustment mechanism. Given the rapid iteration of digital industry technologies, static policy planning struggles to adapt to dynamic transformations. It is advisable to draw inspiration from the EU’s “Smart Specialisation Strategy” [51] to design a dynamic policy adjustment mechanism. Specific operational measures include: (1) establishing multi-party governance platforms, forming a Regional Digital Publishing Strategy Committee involving government, enterprises, research institutes, and industry associations to conduct regular assessments of industry trends and policy impacts; (2) implementing “policy labs” and pilot programs—for emerging policy tools (e.g., AIGC content generation incentives, data asset-based financing support), set up “policy labs” or regulatory sandboxes within designated industrial parks to conduct small-scale trials before large-scale implementation, thereby mitigating policy risks; and (3) building a closed-loop monitoring and evaluation system develop key performance indicators (KPIs) covering industrial innovation, market vitality, and Regional Suitability, and conduct continuous tracking and feedback-based adjustments, thus forming a dynamic governance loop of “monitoring–assessment–adjustment.”

## Supporting information

S1 Appendix Code book of Policy instruments. This appendix presents a code book of policy instruments, which systematically categorizes and quantifies digital publishing policy texts across six dimensions: publishing infrastructure development, copyright governance and protection, content application and scenario promotion, chain shaping and cluster development, financial support, and encouragement of innovative R&D.
(DOCX)
