# Supplementary material for: Research on instrument mix and regional suitability of digital publishing industrial policies: An empirical exploration based on qualitative comparative analysis of fuzzy set
Source: PLoS One. 2026 Apr 13;21(4):e0346245. doi: 10.1371/journal.pone.0346245 (PMC13075672; doi:10.1371/journal.pone.0346245)
Supplement: S1 Appendix — This appendix presents a code book of policy instruments, which systematically categorizes and quantifies digital publishing policy texts across six dimensions: publishing infrastructure development, copyright governance and protection, content application and scenario promotion, chain shaping and cluster development, financial support, and encouragement of innovative R&D. (DOCX) [file pone.0346245.s001.docx]

**Supporting Information**

The minimal dataset underlying the results presented in this study is publicly available in the Figshare repository at [https://doi.org/10.6084/m9.figshare.30343168](https://www.google.com/search?q=https://doi.org/10.6084/m9.figshare.30343168).

We would like to clarify this, which we believe may be a misunderstanding. Our Supporting Information file does not contain any personally identifiable data about ‘human research participants’. The personal data mentioned in the journal's email as requiring protection (e.g. name, address, specific age, contact details, etc.) do not exist in our files at all.

**Appendix 1 Codebook of Policy instruments**

| **Variable Name** | **Operationalisation Definition and Coding Rules** | **Typical Keyword Example** |
| --- | --- | --- |
| ****Publishing Infrastructure Development (SJBSS)**** | Expressions in the policy text involving the provision of basic hardware, platforms or public services for the development of digital publishing. | ‘Digital publishing platform’, ‘Database construction’, ‘Cloud computing centre’, ‘Network infrastructure’, ‘National Laboratory’ |
| ****Copyright Governance and Protection (BQZL)**** | The policy text covers copyright registration, trading, administration, enforcement and anti-piracy, and other expressions aimed at creating a fair market environment. | ‘Copyright Registration’, ‘Copyright Trading Platform’, ‘Combating Internet Infringement and Piracy’, "Intellectual Property Protection ‘, ’Infringement Reporting" |
| ****Content Application and Scenario Promotion (NRYY)**** | Expressions in the policy text that promote the application, consumption and demonstration of digital content products in specific areas (e.g. education, culture and tourism, government). | ‘Digital Reading in Schools’, ‘Intelligent Education’, ‘Digital Cultural Tourism’, ‘Scenario Applications’, ‘Consumption Subsidy’, ‘Demonstration Projects’ |
| ****Chain Shaping and Cluster Development (CYSC)**** | Statements in the policy text encouraging industrial agglomeration, park construction, enterprise cultivation, upstream and downstream industry chain synergy and the creation of industrial clusters. | ‘Industrial parks’, ‘industrial clusters’, ‘leading enterprises’, ‘industry chain synergy’, ‘cluster development’, ‘ecological construction’ |
| ****Financial Support (CZJR)**** | Expressions in the policy text that involve economic incentives such as direct government financial inputs, tax breaks, credit support, and financial guarantees. | ‘Special funds’, ‘Financial subsidies’, ‘Tax incentives’, ‘Loan subsidies’, ‘Financing support’, ‘Venture capital’ |
| ****Encouragement of Innovative R&D (YFGL)**** | Statements in the policy text encouraging research and development, innovation in content, technology, models, etc., as well as the introduction and training of talents. | ‘R&D investment’, ‘technology research’, ‘innovation incentives’, ‘talent training’, ‘Introduction of high-end talents’, ‘Transformation of achievements’ |
